# A Novel Experimental Approach for *In Vivo* Analyses of the Salivary Gland Microvasculature

**DOI:** 10.3389/fimmu.2020.604470

**Published:** 2021-02-17

**Authors:** Bernd Uhl, Constanze Braun, Julian Dominik, Joshua Luft, Martin Canis, Christoph A. Reichel

**Affiliations:** ^1^ Department of Otorhinolaryngology—Head and Neck Surgery, Ludwig-Maximilians-Universität München, Munich, Germany; ^2^ Walter Brendel Centre for Experimental Medicine, Ludwig-Maximilians-Universität München, Munich, Germany

**Keywords:** salivary gland, *in vivo* imaging, microcirculation, leukocyte trafficking, microvascular permeability, inflammation, immunology

## Abstract

Microvascular dysfunction plays a fundamental role in the pathogenesis of salivary gland disorders. Restoring and preserving microvascular integrity might therefore represent a promising strategy for the treatment of these pathologies. The mechanisms underlying microvascular dysfunction in salivary glands, however, are still obscure, partly due to the unavailability of adequate *in vivo* models. Here, we present a novel experimental approach that allows comprehensive *in vivo* analyses of the salivary gland microvasculature in mice. For this purpose, we employed different microscopy techniques including multi-photon *in vivo* microscopy to quantitatively analyze interactions of distinct immune cell subsets in the submandibular gland microvasculature required for their infiltration into the surrounding parenchyma and their effects on microvascular function. Confocal microscopy and multi-channel flow cytometry in tissue sections/homogenates complemented these real-time analyses by determining the molecular phenotype of the participating cells. To this end, we identified key adhesion and signaling molecules that regulate the subset- and tissue-specific trafficking of leukocytes into inflamed glands and control the associated microvascular leakage. Hence, we established an experimental approach that allows *in vivo* analyses of microvascular processes in healthy and diseased salivary glands. This enables us to delineate distinct pathogenetic factors as novel therapeutic targets in salivary gland diseases.

## Introduction

Saliva is an extracellular fluid produced in the head and neck by the paired parotid, submandibular, and sublingual glands as well as by minor salivary glands in the mucosa of lips, tongue, oral cavity, and pharynx (1). A network of tubes and secretory units enables these organs to transport saliva into the oral cavity and pharyngeal tract, which is essential for nutrition (e.g., food digestion, lubrication, and taste) and host defense (as an integral component of the unspecific immune system) (2). Disorders of the salivary glands are based on immunological, bacterial, viral, neoplastic, and iatrogenic etiologies. Importantly, however, these pathologies are all related to inflammatory conditions arising from microvascular dysfunction (3–6).

Upon microvascular injury, white blood cells (leukocytes) start to roll on the luminal aspect of microvascular endothelial cells before they firmly adhere to it and crawl to suitable sites for extravasation. Subsequently, these immune cells pass the endothelial barrier, breach the perivascular basement membrane, and subendothelially locomote through gaps between pericytes to finally migrate through the interstitial tissue to their target destination (7, 8). Neutrophils generally constitute the first leukocyte subset to infiltrate the perivascular tissue, paving the way for other immune cells such as monocytes and lymphocytes (9). The extravasation of leukocytes is associated with enhanced microvascular permeability, which leads to edema formation, reduced oxygenation, and remodeling of the underlying tissue, thus representing hallmarks of the inflammatory response (10). These microvascular processes are critically regulated by parenchymal sentinel cells such as tissue-resident macrophages (TRMs), which maintain microvascular homeostasis in intact and inflamed salivary glands (7, 8, 11–13).

Noteworthy, leukocyte trafficking is highly subset-, tissue-, and stimulus-specific. In many tissues, intravascular rolling of leukocytes is a prerequisite for their subsequent extravasation to the perivascular space. In hepatic sinusoids, renal glomeruli, and central nervous system microvessels, however, immune cells are able to adhere without the need for such loose endothelial cell interactions (14–18). In addition, whereas immune cells use the paracellular route (between endothelial cells) to overcome the endothelial barrier in most tissues, they are supposed to use the transcellular transmigration route in the brain by directly penetrating the endothelial cell body (7, 8). On molecular level, specificity in leukocyte extravasation under certain inflammatory conditions and/or in selected tissues can be explained—at least in part—by differential employment of distinct adhesion/signaling molecules and proteases (e.g., CD62P/P-selectin, CD62E/E-selectin, CD31/PECAM-1, JAM-A, JAM-C, neutrophil elastase, or MMP-9) (19–22). Similarly, the mechanisms regulating microvascular permeability are highly tissue-specific: Mirrored by the differences in their microarchitecture and function, the blood-brain barrier (BBB), for example, is tightly sealed to provide protection, whereas the microvasculature of liver and hormone-synthesizing tissues exhibits relatively large endothelial gaps to allow the release of their products into the systemic circulation (10, 23). In salivary glands, however, the mechanisms underlying the trafficking of leukocytes from the microvasculature to the perivascular tissue as well as the regulation of microvascular permeability remain obscure.

## Materials and Methods

### Animals

Male wild-type (WT) C57BL/6NCrl mice were purchased from Charles River (Sulzfeld, Germany). The experiments of this study were performed with mice at the age of 10 - 15 weeks. Animals were housed under conventional conditions, including food (ssniff, Soest, Germany) and water ad libitum.

### Anesthesia Protocol

Mice were anesthetized using a mixture of ketamine (100 mg/kg body weight) and xylazine (10 mg/kg body weight) administered *via* intraperitoneal (i.p.) injection. Anesthesia was maintained by further i.p. injections of ketamine (100 mg/kg body weight).

### Surgical Implantation of a Catheter in the Left Femoral Artery

In a first step, mice were anesthetized as described above. Subsequently, the anterior skin of the left leg was incised horizontally, the left femoral artery was cannulated in a retrograde manner and a catheter was implanted for administration of antibodies or further reagents, as described elsewhere in detail (24).

### Surgical Preparation of the Submandibular Gland

Anaesthetized mice were cautiously fixed on a custom-built heating plate before the skin of the anterior neck region was disinfected. First, the subcutaneous connective tissue covering the left submandibular gland (synonyms in veterinary anatomy: mandibular gland or submaxillary gland) was exposed *via* a paramedian cervical skin incision. Subsequently, the connective tissue around the left submandibular gland together with the thymus, lymph nodes, and the sublingual gland were carefully detached. For leukocyte trafficking analysis, the internal section embedding the postcapillary venules was exposed by very cautious separation of the salivary gland lobules. The submandibular gland was afterwards placed on a custom-build stage for microscopy as illustrated (**Figure 3**) to stabilize the salivary gland as well as the surgical window to the microcirculation segment of the living organism as a prerequisite for *in vivo* imaging. Throughout the surgical procedure as well as for *in vivo* imaging, the submandibular gland was superfused by warm buffered saline.

### Quantification of Leukocyte Subsets in the Submandibular Gland Homogenates by Multi-Channel Flow Cytometry

In anaesthetized mice, the left submandibular gland was exposed and treated according to the respective experimental protocol. The murine circulation was then cleared from blood by intracardial injection of 10 ml saline (after cutting the *vena cava* for drainage). Finally, the animal was euthanized by cervical dislocation. The salivary gland was then homogenized and the numbers of neutrophils, classical monocytes/monocyte-derived macrophages, non-classical monocytes/tissue-resident macrophages, CD4^+^ T lymphocytes, CD8^+^ T lymphocytes, and B lymphocytes were analyzed in the cell samples by an automated cell counter in combination with multi-channel flow cytometry (Gallios, Beckman Coulter Inc, Brea, California USA). Myeloid leukocytes were identified as CD45^pos^ CD11b^pos^ cells, which were further differentiated into neutrophils (GR-1^high^, CD115^neg^ cells), classical monocytes/monocyte-derived macrophages (GR-1^high^, CD115^pos^ cells), and non-classical monocytes/tissue-resident macrophages (GR-1^low^, CD115^pos^ cells). Non-myeloid leukocytes (CD45^pos^ CD11b^neg^ cells) were then discriminated into CD4^+^ T lymphocytes (CD4^pos^), CD8^+^ T lymphocytes (CD8a^pos^), and B lymphocytes (CD19^pos^), as described elsewhere (25, 26).

### Immunohistochemistry

In anaesthetized mice, the left submandibular gland was exposed and treated according to the respective experimental protocol. Harvested submandibular glands were embedded in OCT (TissueTec) and frozen, before they were sectioned (thickness of 10 μm) on a cryostat (Leica). Sections were fixed in methanol, incubated with Alexa-Fluor 633 labeled antibody against CD31/PECAM-1 as well as one of the following antibodies each: R-PE labeled antibodies against CD62P/P-selectin, CD54/ICAM-1, or CD106/VCAM-1 overnight at 4°C. The sections were then imaged using a Leica SP5 confocal laser-scanning microscope (Leica Microsys-tems, Wetzlar, Germany) with an oil-immersion lens (Leica; 63x; NA 1.40), as described elsewhere (27). Confocal z-stacks of 10 µm (z-spacing 0.5μm) were acquired. To determine the expression of the adhesion and signaling molecules on the microvascular endothelium (identified by the CD31/PECAM-1 expression), the fluorescence levels of the R-PE labeled antibodies were analyzed offline in Z-projections of the stacks using the software Fiji (distribution of the software ImageJ) (28).

### *In Vivo* Epifluorescence Microscopy (Leukocyte Trafficking)

In anaesthetized mice, the left submandibular gland was exposed and treated according to the respective experimental protocol. In order to analyze leukocyte trafficking in the submandibular gland, imaging of the postcapillary venule section in the salivary gland was a prerequisite, as mentioned above.

The setup for *in vivo* microscopy was centered around an Axio Scope.A1 microscope equipped with a Colibiri 2 LED light source (Carl Zeiss Microscopy GmbH, Göttingen, Germany) for fluorescence epi-illumination microscopy, as described earlier (26). The vasculature in the submandibular gland was visualized by systemic administration of high-molecular Fluorescein isothiocyanate (FITC) dextran (average mol wt 2,000,000; Sigma-Aldrich, Taufkirchen, Germany). To differentiate neutrophils (GR-1^high^, CD115^neg^) from classical (GR-1^high^, CD115^pos^) and non-classical monocytes (GR-1^low^, CD115^pos^), intravascular leukocytes were labeled *via* systemic administration of fluorescently labeled monoclonal antibodies against mouse GR-1 (clone RB6-8C5) and mouse CD115 (CSF-1R; clone AFS98). LED-light was used for excitation (470 nm, 555 nm, and 625 nm) and a QUAD filter set (QUAD DAPI (4′,6-diamidino-2-phenylindole)/FITC/Cy3/Cy5 sbx HC Filterset; AHF Analysentechnik AG, Tübingen, Germany) was employed for discrimination of the fluorescent emissions.

The software Fiji (distribution of the software ImageJ) was used for offline analysis of the images and videos recorded during microscopy to quantify leukocyte trafficking (28). Leukocytes were identified by their fluorescence signal (see above). Rolling leukocytes were defined as cells moving slower than the intravascular blood flow and quantified as rolling cells passing a defined vessel section within 60 s. Rolling velocity was calculated by measuring the distance covered by endothelially rolling leukocytes within 10 s. Firmly adherent leukocytes were determined as those cells resting in the associated blood flow for more than 30 s at the length of 100 µm as related to the luminal surface of the vessel during 60 s.

### *In Vivo* Multi-Photon Microscopy (Microvascular Permeability)

In anaesthetized mice, the left submandibular gland was exposed and treated according to the respective experimental protocol. Microvascular permeability was determined by measuring microvascular extravasation of the macromolecule FITC dextran (MW 500,000, Sigma Aldrich), which was administered *via* an intraarterial catheter (4 ml per kg body weight of 50 mg FITC dextran diluted in 1 ml saline) and investigated 30 min later in six microvascular segments with venules of the submandibular gland. An upright TriMScope multi-photon microscope (LaVisionBiotec, Bielefeld, Germany) in combination with an Olympus XLUMP-lanFl 20×/0.95 W dipping objective was used for imaging of the submandibular gland similar as described elsewhere in detail (24). FITC-labeled dextran (average mol wt 500,000; Sigma-Aldrich, Taufkirchen, Germany) was excited by the pulsed laser at the wavelength of 800 nm and its fluorescence signal was detected at the wavelength range of 525 ± 25 nm. Mean gray values of the fluorescent signal of the FITC dextran were measured by digital image analysis (Fiji software/ImageJ distribution) in six randomly selected regions of interest (ROIs; 50x50 μm²) in the perivascular tissue of each microvascular segment, which were localized 25 μm distant from the postcapillary venule under investigation (28). Afterwards, the average of the mean gray values of the different segments was calculated.

### Experimental Groups

Animals were assigned randomly to the following groups: In a first set of experiments, trafficking of leukocytes (n = 6) and/or endothelial expression profiles of distinct adhesion and signaling molecules (n = 4) were analyzed in submandibular glands of wildtype mice, which were either superfused with tumor necrosis factor (TNF) in saline (1 µg/ml or 0.5 ng/ml) or saline only. In addition, the single steps of the leukocyte extravasation cascade were examined for neutrophils and classical monocytes similarly in submandibular glands of WT mice superfused with tumor necrosis factor (TNF) in saline (1 µg/ml) or saline only. The mechanisms were further characterized in TNF-stimulated submandibular glands of WT mice treated systemically either with a blocking anti-CD62P/P-selectin-antibody (50 µg), a blocking anti-CD54/ICAM-1-antibody (50 µg), a depleting anti-Ly6G-antibody (neutrophil depletion; 2x 50 µg), or the respective isotype controls (50 µg; n = 4–6). In further experiments, microvascular permeability was studied in submandibular glands superfused with TNF in saline (1 µg/ml) or saline only of WT mice receiving either a depleting anti-Ly6G-antibody (neutrophil depletion; 2x 50 µg) or the respective isotype controls (2x 50 µg; n = 6). The experiments were performed during working hours between 8:00 a.m. and 6:00 p.m in the operating room of the institute.

### Experimental Protocols

To study the TNF-mediated trafficking of different leukocyte subpopulations or microvascular permeability, submandibular glands of anesthetized WT mice receiving distinct blocking, depleting, or isotype control antibodies according to the respective experimental group were superfused with TNF in saline (1 µg/ml) or saline only for 5 h. In one group of experiments, submandibular glands were homogenized to determine the cell numbers of different leukocyte subsets recruited to the organ *via* an automated cell counter (ProCyte Dx Hematology Analyzer, IDEXX Laboratories, Inc., Maine, USA) and multi-channel flow cytometry. In a further experimental group, the single steps of the leukocyte recruitment cascade were analyzed by epifluorescence *in vivo* microscopy of submandibular glands in mice, which received fluorescence-labeled antibodies *via* the tail vein for the differentiation of neutrophils, classical and non-classical monocytes 10 min prior to imaging. In another set of experiments, the extravasation of systemically administered Alexa Fluor 488-labeled dextran (500.000 Da) was assessed 30 min after i.v. injection *via* multi-photon *in vivo* fluorescence microscopy in the perivascular tissue of salivary glands as a parameter for microvascular permeability.

### Inhibitors and Antibodies

For stimulation of submandibular glands, recombinant mouse TNF protein (aa 80–235; R&D Systems, Minneapolis, USA) was used.

To differentiate the recruited leukocyte subsets *via* flow cytometry, the following fluorescently labeled antibodies were used: APC/Cyanine7 monoclonal anti-mouse anti-CD45 antibody (clone: 30-F11; BioLegend, San Diego, USA), FITC monoclonal anti-mouse anti-CD11b antibody (clone: M1/70; Biolegend, San Diego, USA), PE monoclonal anti-mouse Ly-6G/Ly-6C (GR-1) antibody (clone: RB6-8C5; Biolegend, San Diego, USA), eFluor450 monoclonal anti-mouse anti-F4/80 antibody (clone: BM8, ThermoFisher Scientific, Waltham, USA), PerCP-Cyanine5.5 monoclonal anti-mouse anti-CD19 antibody (clone: eBio1D3 (1D3), ThermoFisher Scientific, Waltham, USA), Alexa Fluor 700 monoclonal anti-mouse anti-CD4 antibody (clone: GK1.5, ThermoFisher Scientific, Waltham, USA), and PE-Cyanine7 monoclonal anti-mouse anti-CD8a antibody (clone: 53-6.7, ThermoFisher Scientific, Waltham, USA).

In the *in vivo* model, an Alexa Fluor^®^ 594 monoclonal anti-mouse CD115 (CSF-1R) antibody (clone: AFS98; BioLegend, San Diego, USA) and an Alexa Fluor^®^ 647 monoclonal anti-mouse Ly-6G/Ly-6C (Gr-1) antibody (clone: RB6-8C5; BioLegend, San Diego, USA) were employed to identify neutrophils as well as classical and non-classical monocytes. FITC dextran (average mol wt 2,000,000; Sigma-Aldrich, Taufkirchen, Germany) was used to visualize the microvessels in the salivary gland.

Multi-photon microscopy was employed on submandibular glands of mice after systemic administration of FITC dextran (average mol wt 500,000; Sigma-Aldrich, Taufkirchen, Germany) to examine the microvascular permeability.

A purified blocking monoclonal anti-mouse anti-CD54 antibody (clone: YN1/1.7.4; BioLegend, San Diego, USA) and a purified blocking monocloncal anti-mouse/rat anti-CD62P/P-selectin Antibody (clone: RMP-1, BioLegend, San Diego, USA) were administered systemically to block the respective receptors. Depletion of neutrophils in mice *in vivo* (**Supplementary Figure 2**) was achieved by systemic administration of a depleting monoclonal anti-mouse anti-Ly6G-antibody (clone: 1A8; Bio X Cell, West Lebanon, USA).

In the immunohistochemistry assay, the following antibodies were used: An Alexa-Fluor 633 labeled monoclonal anti-mouse anti-CD31/PECAM-1 antibody (clone MEC13.3, Biolegend, San Diego, USA) for detection of the microvascular endothelium. Co-Staining with a R-phycoerythrin labeled monoclonal anti-mouse anti-CD62P/P-selectin antibody (clone RMP-1, Biolegend, San Diego, USA), a R-phycoerythrin labeled monoclonal anti-mouse anti-CD54/ICAM-1 antibody (clone YN1/1.7.4, Biolegend, San Diego, USA), or a R-phycoerythrin labeled monoclonal anti-mouse anti-CD106/VCAM-1 antibody (clone 429, Biolegend, San Diego, USA) allowed the analysis of the endothelial expression on microvessels in the submandibular glands.

### Statistics

Data analysis was performed with a statistical software package (SigmaPlot; Systat Software GmbH, Erkrath, Germany) employing the Mann-Whitney rank-sum test (two groups) or analysis of variance on ranks test, followed by the Dunnett test (>2 groups) for the estimation of stochastic probability in intergroup comparisons. Mean values and standard error of the mean are given. P values < 0.05 were considered significant.

### Study Approval

All animal experiments were approved by the local governmental authorities (Government of Upper Bavaria; “Regierung von Oberbayern”) and conducted according to the guidelines to ensure animal welfare. The manuscript follows the ARRIVE (Animal Research: Reporting of *In Vivo* Experiments) guidelines for reporting on animal studies.

## Results

### TNF Promotes Subset-Specific Leukocyte Trafficking to Salivary Glands

**Figure 1:**
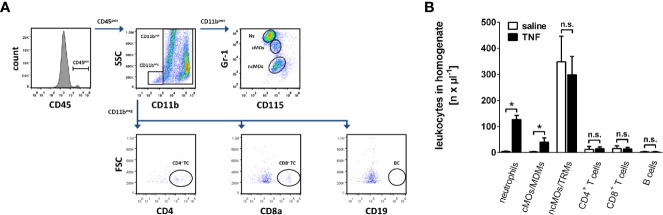
Trafficking of leukocytes into the inflamed submandibular gland. **(A)** The gating strategy for the differentiation of neutrophils, classical monocytes (cMOs)/monocyte-derived macrophages (MDMs) and non-classical monocytes (ncMOs)/tissue-resident macrophages (TRMs), CD4^+^ and CD8^+^ T lymphocytes, as well as B lymphocytes in homogenates of murine submandibular glands in multi-channel flow cytometry analyses. **(B)** Quantitative data on the recruitment of these leukocyte subsets into submandibular glands upon superfusion with recombinant mouse TNF (1 µg/ml; 5 h) or saline (mean ± SEM for n = 6; *p < .05 vs. saline; n.s., non significant).

Leukocyte recruitment from the microvasculature to the perivascular tissue is a key event in the inflammatory response (7, 8). In a first set of experiments, we therefore sought to analyze the trafficking of major leukocyte subsets including neutrophils, classical monocytes and non-classical monocytes, CD4^+^ and CD8^+^ T lymphocytes, as well as B lymphocytes to mouse submandibular glands under homeostatic and inflammatory conditions. TNF (Tumor necrosis factor) was used as an inflammatory stimulus since it represents one of the most prominent pro-inflammatory cytokines present in various inflammatory disorders including salivary gland pathologies (29, 30). In gland homogenates of unstimulated animals (superfused with saline only), low numbers of neutrophils, classical monocytes/monocyte-derived macrophages (MDMs), CD4^+^ T lymphocytes, CD8^+^ T lymphocytes, and B lymphocytes were found by multi-channel flow cytometry, whereas numbers of non-classical monocytes/tissue-resident macrophages (TRMs) were significantly higher (**Figures 1A, B**; n = 6). Superfusion of the glands with TNF in a very low dose of 0.5 ng per ml saline, which is comparable to TNF levels in blood or saliva of humans with chronically inflamed salivary glands (31), did not significantly alter the number of leukocytes in homogenized submandibular glands (**Supplementary Figure 1**, n = 6). In contrast, superfusion with TNF in the dose of 1 µg per ml saline, a dose employed for analyzing the effect of TNF on leukocyte trafficking in various organ models (32–34), predominantly induced infiltration of neutrophils and classical monocytes/monocyte-derived macrophages to the inflamed tissue, whereas the numbers of non-classical monocytes/tissue-resident macrophages, CD4^+^ and CD8^+^ T lymphocytes, or B lymphocytes remained unaltered (**Figure 1B**; n = 6). In line with a previous study (35), these dose-dependent effects of TNF indicate that saliva or systemic blood levels of the TNF protein do not directly mirror (local) intraparenchymal TNF protein levels required for the induction of microvascular inflammatory processes such as leukocyte trafficking. Thus, TNF dose-dependently promotes the recruitment of neutrophils and classical monocytes/monocyte-derived macrophages to salivary glands.

### TNF Induces the Expression of Distinct Adhesion and Signaling Molecules on the Microvascular Endothelium of Salivary Glands

**Figure 2:**
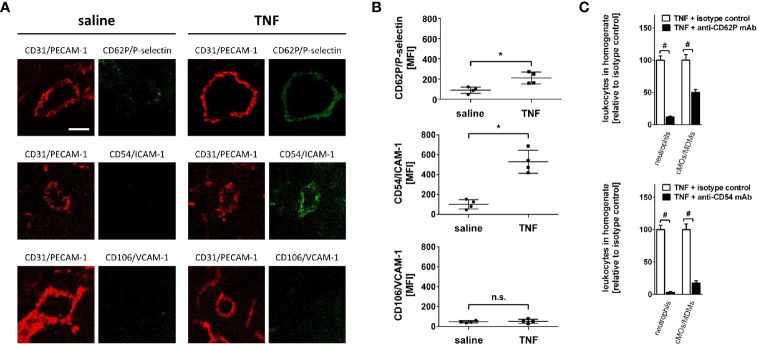
Mechanisms of leukocyte trafficking into the inflamed submandibular gland. **(A)** Representative microscopy images (z-projections) of immunohistochemically stained tissue sections of control (superfusion with saline) and inflamed (superfusion with TNF) submandibular glands illustrating the expression of CD62P/P-selectin, CD54/ICAM-1, and CD106/VCAM-1 (green) in CD31/PECAM-1 (red)-positive postcapillary venules (scale bar: 10 µm). **(B)** Quantitative data for endothelial expression of CD62P/P-selectin, CD54/ICAM-1, and CD106/VCAM-1 of submandibular glands superfused with TNF or saline (mean ± SEM for n = 4; *p < .05 vs. saline; n.s., not significant). **(C)** Quantitative data for TNF-induced recruitment of neutrophils and classical monocytes (cMOs)/monocyte-derived macrophages (MDMs) to submandibular glands in animals treated with blocking monoclonal anti-CD62P/P-selectin or anti-CD54/ICAM-1 antibodies or isotype control antibodies (mean ± SEM for n = 6; ^#^p < .05 vs. isotype control).

Extravasation of leukocytes is mediated by interactions of distinct adhesion and signaling molecules on leukocytes and the microvascular endothelium. Importantly, these molecular signatures on endothelial cells are highly tissue-specific (8, 19, 22). To evaluate the expression of key adhesion and signaling molecules on the microvascular endothelium of salivary glands, we performed immunostaining and confocal microscopy analyses of gland cryosections. In unstimulated animals, surface expression of CD62P/P-selectin, CD54/ICAM-1, or CD106/VCAM-1 was low on CD31/PECAM-1-positive microvascular endothelial cells. In contrast, stimulation with TNF induced a significant increase in the endothelial expression of CD62P/P-selectin and CD54/ICAM-1 as compared to controls, whereas the endothelial expression of CD106/VCAM-1 remained unchanged (**Figures 2A, B**; n = 4). Our results corroborate previous findings demonstrating a multiplication of the expression levels of CD62P/P-selectin or CD54/ICAM-1 after 5 h of TNF stimulation in different organs (36). Hence, TNF induces the expression of distinct adhesion and signaling molecules in the salivary gland microvasculature.

### TNF Promotes the Trafficking of Neutrophils and Classical Monocytes/Monocyte-Derived Macrophages to Salivary Glands *via* CD62P/P-Selectin and CD54/ICAM-1

Since TNF increased the expression of CD62P/P-selectin and CD54/ICAM-1 on the microvascular endothelium, we hypothesized that these molecules are functionally relevant for the trafficking of neutrophils and classical monocytes/monocyte-derived macrophages to submandibular glands. Confirming this hypothesis, antibody-blockade of CD62P/P-selectin or CD54/ICAM-1 significantly reduced the TNF-elicited recruitment of neutrophils and classical monocytes/monocyte-derived macrophages to salivary glands as compared to isotype control antibody-treated animals. Specifically, the reduction in these myeloid leukocyte responses was significantly more pronounced upon blockade of CD54/ICAM-1 than upon blockade of CD62P/P-selectin (**Figure 2C**; n = 6), indicating that TNF involves particularly CD54/ICAM-1 and—to a lesser extent—CD62P/P-selectin to promote the trafficking of neutrophils and classical monocytes/monocyte-derived macrophages to salivary glands.

### TNF-Elicited Intravascular Rolling and Firm Adherence of Myeloid Leukocytes in Postcapillary Venules of Salivary Glands Is Mediated by CD62P/P-Selectin and CD54/ICAM-1

**Figure 3:**
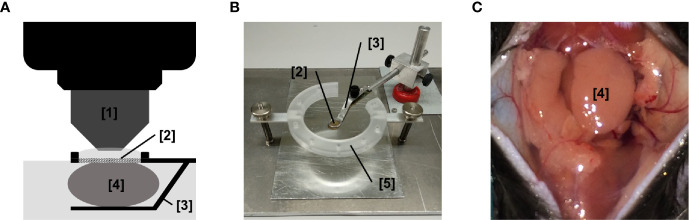
Experimental setup for *in vivo* microscopy analyses of the submandibular gland microvasculature. **(A)** Schematic illustration of the microscopy setup for *in vivo* analysis of the microcirculation in the mouse submandibular gland. **(B)** Representative image of the microscopy stage for *in vivo* imaging of the microcirculation in the mouse submandibular gland. **(C)** Representative image of the operation situs of the anterior neck region after careful dissection of the submandibular gland. ([1] dipping objective; [2] glass-window for microscopy; [3] mounting of stage; [4] submandibular gland (synonyms in veterinary anatomy: mandibular gland or submaxillary gland); [5] fixation ring for skin sutures; n.s., non significant.

Leukocyte recruitment from the microvasculature to the perivascular tissue is a highly subset- and tissue-dependent process that includes a sequential, multi-step cascade of leukocyte-endothelium interactions (14–16). To analyze the single steps of the extravasation process of leukocytes to salivary glands, we established a novel experimental approach for multi-channel *in vivo* microscopy analyses of the salivary gland microvasculature (**Figure 3**).

**Figure 4:**
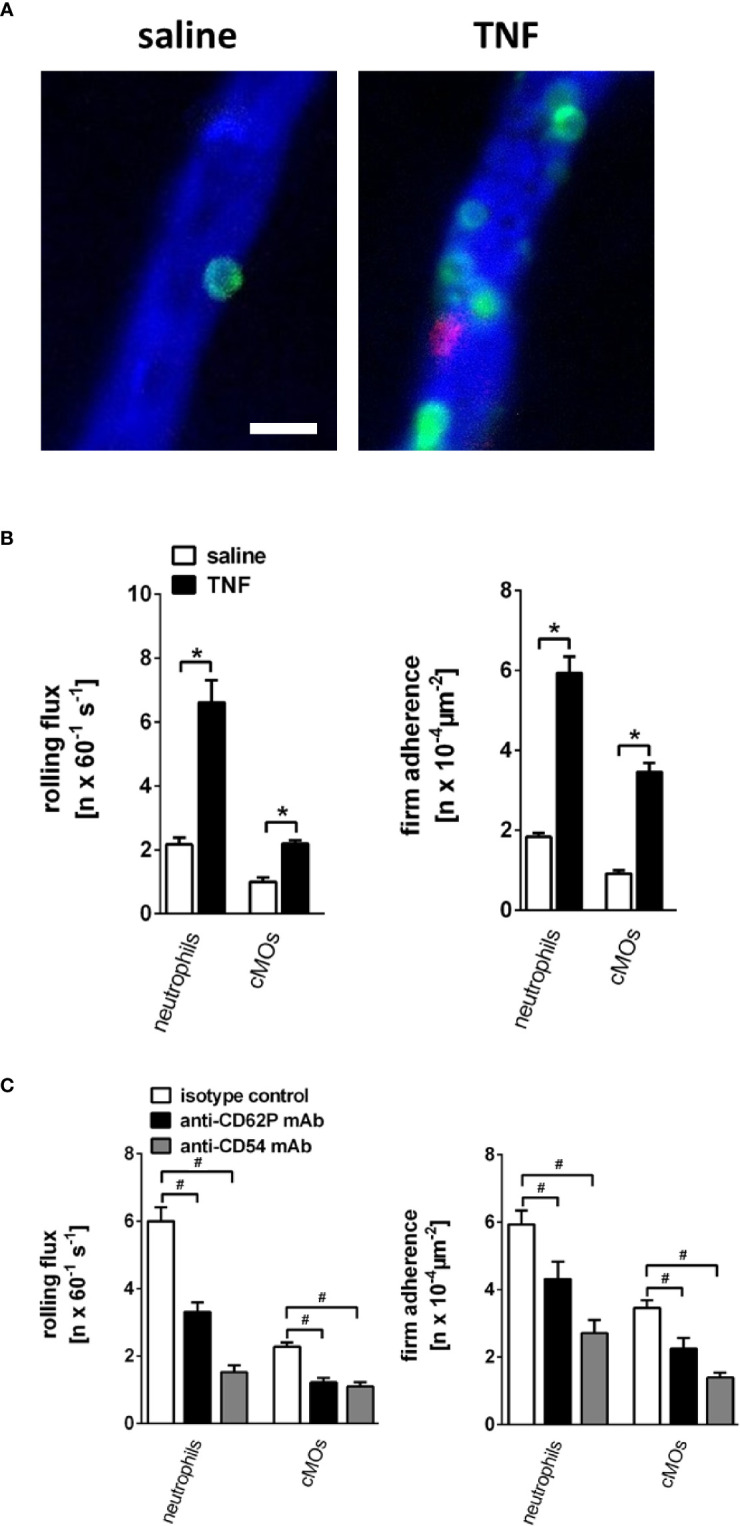
Microvascular leukocyte-endothelial cell interactions in the inflamed submandibular gland. **(A)** Representative multi-channel epifluorescence *in vivo* microscopy images illustrating postcapillary venules in control (superfusion with saline) and inflamed (superfusion with TNF) submandibular glands (scale bar: 20 µm; intravascular FITC dextran in blue, Ly-6G/Ly6C+ cells (GR-1) in green, and CD115+ cells in red). **(B)** Quantitative data for intravascular rolling flux and firm adherence of neutrophils and classical monocytes (cMOs) in postcapillary venules of submandibular glands superfused with TNF or saline (mean ± SEM for n = 4; *p < .05 vs. saline). **(C)** Quantitative data for TNF-induced intravascular rolling flux and firm adherence of neutrophils and cMOs in postcapillary venules of submandibular glands in animals treated with blocking monoclonal anti-CD62P/P-selectin antibodies or anti-CD54/ICAM-1 antibodies as compared to isotype control antibody-treated animals (mean ± SEM for n = 4; ^#^p < .05 vs. isotype control).

Under unstimulated conditions, numbers of intravascularly rolling as well as adherent neutrophils and classical monocytes in gland microvessels were found to be low. In contrast, stimulation with TNF induced a significant elevation in the numbers of intravascular rolling as well as firmly adherent neutrophils and classical monocytes in postcapillary venules but not in capillaries or arterioles of the gland (**Figures 4A, B**; n = 4). This elevation in numbers of intravascularly rolling and firmly adherent neutrophils and classical monocytes was significantly diminished upon antibody-blockade of CD62P/P-selectin or CD54/ICAM-1 (**Figure 4C**; n = 4). Conversely, the rolling velocity of neutrophils and classical monocytes was reduced in TNF-stimulated animals (neutrophils: 17.8 µm/s; cMOs: 12.0 µm/s) as compared to unstimulated controls (neutrophils: 26.9 µm/s; cMOs: 25.2 µm/s). Blockade of CD54/ICAM-1 (neutrophils: 5.2 µm/s; cMOs: 1.9 µm/s), but not of CD62P/P-selectin (neutrophils: 14.3 µm/s; cMOs: 10.6 µm/s), even further diminished the decreased rolling velocity of the very few remaining rolling leukocytes upon stimulation with TNF. In summary, TNF-elicited rolling and firm adherence of myeloid leukocytes in postcapillary venules of salivary glands is mediated by CD62P/P-selectin and CD54/ICAM-1.

### Neutrophils Support the Trafficking of Classical Monocytes to Inflamed Salivary Glands

**Figure 5:**
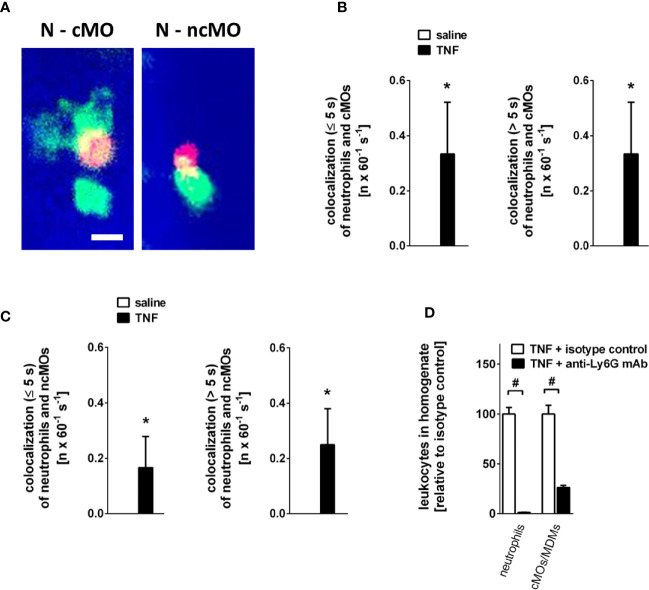
Intravascular interactions between leukocytes in the inflamed submandibular gland. **(A)** Representative multi-channel epifluorescence *in vivo* microscopy images illustrating interactions between neutrophils (N) and classical monocytes (cMO) or non-classical monocytes (ncMO) in postcapillary venules of TNF-stimulated submandibular glands (scale bar: 10 µm; intravascular FITC dextran in blue, Ly-6G/Ly6C+ cells (GR-1) in green, and CD115+ cells in red). **(B, C)** Quantitative data for short (≤ 5s) and long (> 5s) interactions of neutrophils and classical monocytes (cMOs) **(B)** or of neutrophils and non-classical monocytes (ncMOs) **(C)** in postcapillary venules of submandibular glands upon superfusion of TNF or saline (mean ± SEM for n = 4; *p < .05 vs. saline). **(D)** Quantitative data for TNF-elicited recruitment of neutrophils and classical monocytes (cMOs)/monocyte-derived macrophages (MDMs) to submandibular glands in animals treated with neutrophil-depleting anti-Ly6G or isotype control antibodies (mean ± SEM for n = 6; ^#^p < .05 vs. isotype control).

Intravascular interactions of leukocytes among each other support their extravasation to the perivascular tissue and thus are essential for an effective inflammatory response (37). Under unstimulated conditions, interactions between neutrophils and classical or non-classical monocytes were not detectable in the gland microvasculature. Stimulation with TNF, however, induced both incidental (<5 s) and intense (>5 s) intravascular interactions of neutrophils with classical and—to a lesser degree—with non-classical monocytes (**Figures 5A–C**; n = 4).

Neutrophils contribute to the recruitment of monocytes in many tissues (38). To characterize the role of neutrophils for TNF-elicited classical monocyte trafficking in salivary glands, we performed experiments in neutropenic animals. Here, we found that antibody-mediated depletion of neutrophils (**Supplementary Figure 2**; n = 3) significantly diminished the number of classical monocytes/monocyte-derived macrophages recruited to TNF-stimulated glands as compared to isotype control antibody-treated animals (**Figure 5D**; n = 6), suggesting that neutrophils pave the way to salivary glands for classical monocytes/monocyte-derived macrophages.

### Neutrophils Promote Microvascular Hyperpermeability in Inflamed Salivary Glands

**Figure 6:**
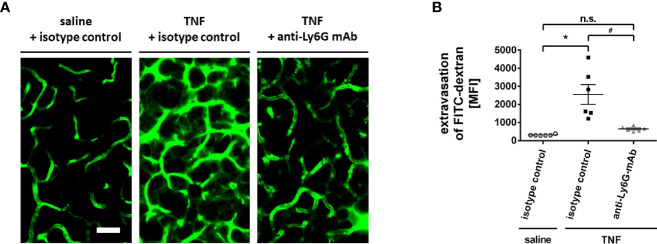
Role of neutrophils for microvascular hyperpermeability in the inflamed submandibular gland. **(A)** Representative multi-photon *in vivo* microscopy images illustrating sections of the microvasculature in control (superfusion with saline) and inflamed (superfusion with TNF) submandibular glands treated with isotype control or neutrophil-depleting anti-Ly6G antibodies (scale bar: 50 µm; FITC dextran in green). Quantitative data is shown in **(B)**; mean ± SEM for n = 6; *p < .05 vs. isotype control + saline; ^#^p < .05 vs. isotype control + TNF; n.s., non significant).

In addition to leukocyte trafficking, microvascular hyperpermeability represents a hallmark of the inflammatory response (39). As a measure of microvascular permeability, the leakage of the macromolecule FITC dextran (500.000 kDa) into the perivascular tissue of the mouse submandibular gland microvasculature was analyzed by multi-photon *in vivo* microscopy. Under unstimulated conditions, microvascular leakage of FITC dextran to the perivascular tissue was minimal. Stimulation with TNF, however, led to a significant extravasation of this macromolecule to the perivascular space (**Figures 6A, B**; n = 6).

Since neutrophils critically modulate microvascular permeability in different tissues (40), we subsequently sought to elucidate the functional relevance of these immune cells for TNF-dependent hyperpermeability in salivary glands. In our experiments, the TNF-elicited elevation in the leakage of FITC dextran to the gland’s parenchyma was significantly reduced in neutropenic animals (**Figures 6A, B**; n = 6). Thus, neutrophils promote hyperpermeability in inflamed salivary glands.

## Discussion

Although numerous pathological conditions (e.g., bacterial and viral infections, autoimmune diseases such as Sjögren’s syndrome, malignant tumors, or irradiation) may cause disorders of the head neck salivary glands, these diseases are all characterized by acute or chronic inflammatory changes arising from microvascular dysfunction (1, 3–6, 41). Immune cell accumulation in microvasculature and perivascular tissue as well as microvascular leakage are key components of inflammatory reactions (8, 10) that are regulated by parenchymal sentinel cells such as tissue-resident macrophages (7, 8, 11–13). Targeting these events in the injured gland microvasculature might therefore represent a promising approach for the prevention and treatment of these pathologies. The mechanisms controlling such inflammatory processes in salivary glands, however, are still poorly understood (9, 14).

Innate immune cells such as neutrophils and monocytes serve as first responders in inflammatory reactions, unspecifically eliminating invading pathogens and injured cells. As effector cells of the adaptive immune system, lymphocytes subsequently employ more specific cellular and humoral mechanisms to further enforce host defense (42). Previous experimental approaches granted insights into immune cell responses in superficial parenchymal regions of salivary glands (12, 43–45) but did not allow the visualization and quantitative analysis of the underlying processes in the microvasculature deep inside the gland’s parenchyma (7, 8). In the present study, we therefore combined diverse experimental approaches to explore microcirculatory events in salivary glands. Employing multi-channel flow cytometry on tissue homogenates of murine submandibular glands (synonyms in veterinary anatomy: mandibular gland or submaxillary gland), we first sought to basically analyze the trafficking of immune cells to the gland parenchyma (46, 47). Gland inflammation was induced by superfusion of the cytokine TNF, which represents one of the most prominent inflammatory mediators in salivary gland pathologies (29, 30). In these experiments, we found that predominantly neutrophils and classical monocytes/monocyte-derived macrophages accumulate in the inflamed tissue, whereas responses of non-classical monocytes/tissue-resident macrophages, CD4^+^ and CD8^+^ T lymphocytes, or B lymphocytes remained absent. These observations extend previous findings in different tissues corroborating TNF as potent, mediator of myeloid leukocyte trafficking. Interestingly, blockade of TNF has recently been reported to attenuate lymphocyte responses in Sjögren’s-like sialadenitis and other pathologies, pointing to indirect effects of this cytokine on these adaptive immune cells. In this regard, neutrophils and classical monocytes/monocyte-derived macrophages have been demonstrated to modulate activation, proliferation, and recruitment of lymphocytes either in a stimulating [e.g., function as antigen presenting cell, T-cell activation *via* the microbial metabolite (E)-4-hydroxy-3-methyl-but-2-enyl pyrophosphate (HMB-PP)] or an inhibiting manner [e.g., by release of reactive oxygen species (ROS), arginase-I (ARG), and TGF-beta, or by expression of PD-L1] (29, 48, 49).

Extravasation of immune cells from the microvasculature to the perivascular tissue is a highly regulated multi-step process that requires a complex interplay of distinct adhesion and signaling molecules on leukocytes and on microvascular endothelial cells. In many tissues, activated endothelial cells express selectins on their luminal surface to capture circulating leukocytes and to slow them down while rolling on the endothelium. This enables endothelially expressed members of the immunoglobulin superfamily (e.g., CD54/ICAM-1, CD106/VCAM-1) to interact with leukocyte integrins, ultimately facilitating intravascular adherence and subsequent transmigration of these immune cells into the perivascular parenchyma. Importantly, the endothelial expression of these molecules and their functional relevance for leukocyte trafficking is highly tissue-specific (8, 19, 22). Using immunostaining and confocal microscopy on tissue cryosections (26, 27), surface levels of CD62P/P-selectin and CD54/ICAM-1, but not of CD106/VCAM-1, were significantly enhanced on microvascular endothelial cells of inflamed glands as compared to unstimulated controls. Functional experiments further revealed that CD54/ICAM-1 and—to a lesser extent—CD62P/P-selectin promote the trafficking of neutrophils and classical monocytes/monocyte-derived macrophages to the gland’s parenchyma, emphasizing the major role of these adhesion and signaling molecules for the regulation of myeloid leukocyte responses. Although CD62P/P-selectin and CD54/ICAM-1 are required for leukocyte infiltration of liver, kidney, lung, brain, or peritoneum (50, 51), these molecules are dispensable for immune cell trafficking under specific inflammatory conditions such experimental autoimmune encephalomyelitis or lymphocyte transmigration into secondary lymphoid organs (15, 52). Conversely, CD106/VCAM-1 is not required for leukocyte responses in chronic vasculitis but is critical, e.g., for fine ambient particle matter-induced lung injury (53, 54).

To further analyze the single steps of the extravasation process of different immune cells, we established a novel experimental approach that allows *in vivo* analyses of the salivary gland microvasculature. For this purpose, we gently dissected the different lobes of the submandibular gland in anesthetized mice to gain access to the microvasculature deep inside the gland’s parenchyma. Subsequently, endothelial interactions of blood cells were identified by *in vivo* immunostaining and multi-channel *in vivo* microscopy. In unstimulated gland microvessels, only few intravascular rolling and adherent leukocytes were observed, most probably due to a minimal surgical trauma as reported in different experimental models such as the cremaster muscle or the mesentery (55, 56). Consistent with our previous findings in tissue homogenates, induction of inflammation by TNF, however, induced a strong increase in intravascular rolling and firm adherence of neutrophils as well as classical monocytes in postcapillary venules but not in capillaries or arterioles. This intravascular accumulation of myeloid leukocytes required CD62P/P-selectin and CD54/ICAM-1 (7, 8). Mechanistically, selectins such as CD62P/P-selectin mediate molecular interactions characterized by rapid association/dissociation rates, thus allowing them to initiate the initial contact and the subsequent high velocity rolling of leukocytes on the endothelium under shear. In contrast, adhesion molecules of the immunoglobulin superfamily including CD54/ICAM-1 recognize specific integrins on the surface of rolling leukocytes, hence promoting more stable interactions of immune cells with microvascular endothelial cells (7, 8). Accordingly, rolling neutrophils and classical monocytes demonstrated a reduced rolling velocity in inflamed submandibular glands as compared to unstimulated controls. The rolling velocity of the few remaining rolling myeloid leukocytes in animals treated with blocking CD62P/P-selectin-antibodies remained low, whereas the rolling velocity of the even fewer, sporadically rolling neutrophils and classical monocytes in animals treated with blocking CD54/ICAM-1-antibodies further decreased. Consequently, our data suggest that CD54/ICAM-1 represents as a key regulator of myeloid leukocyte responses in the inflamed salivary gland microvasculature.

In addition to their interplay with endothelial cells, immune cells interact with each other during their extravasation to the perivascular tissue (37). In the microvasculature of unstimulated glands, interactions of neutrophils and monocytes were absent. Upon onset of inflammation, however, loose and, later, more intense interactions of intravascularly accumulating neutrophils and monocytes gradually increased. These events particularly supported the extravasation of classical monocytes/monocyte-derived macrophages since infiltration of inflamed glands with these immune cells was severely compromised in neutropenic animals. These findings extend previous reports of neutrophil-driven extravasation of classical monocytes in peritoneum, lung, and brain. Here, it has been shown that neutrophils deposit released granule proteins (e.g., CCL3/MIP-1α, CCL2/MCP-1, LL-37) and—in advanced stages of disease—’find me’ and ‘eat me’ signals on the microvascular endothelium and in the parenchyma, thus promoting intravascular adhesion and subsequent extravasation of classical monocytes (57).

Beyond immune cell trafficking, enhanced microvascular permeability is a hallmark of the inflammatory response, ultimately leading to edema formation, remodeling, and reduced oxygenation of the affected tissue (10). To measure microvascular leakage in salivary glands, we employed multi-photon microscopy to determine the extravasation of intravenously applied FITC-labeled dextran from postcapillary venules in real time. Importantly, the salivary gland microvasculature supports the formation of primary saliva *via* a basal, physiological permeability for water and small molecules (<70 kDa), a process regulated by tight junctions between microvascular endothelial cells (58). To measure hyperpermeability in inflamed salivary glands, we therefore used high molecular weight FITC-labeled dextran molecules (500 kDa) in our experiments. Accordingly, no leakage of FITC dextran to the perivascular space was observed under unstimulated conditions. Induction of inflammation, however, strongly enhanced gland microvascular permeability. This process was critically dependent on the presence of neutrophils as evidenced in neutropenic animals. On molecular level, these immune cells promote endothelial permeability *via* secretion of chemokines (e.g., CXCL 1, 2, 3, and 8), arachidonic acid, ATP, or adhesion-dependent processes such as integrins interacting with endothelial adhesion and signaling molecules (e.g., CD54/ICAM-1) (40). In summary, our experimental data indicate that TNF promotes micro- and perivascular accumulation of myeloid leukocytes as well as subsequent microvascular hyperpermeability in salivary glands. Consequently, the previously reported beneficial effects of pharmacological TNF blockade on salivary gland function in autoimmune Sjögren’s-like sialadenitis (29, 48) might be—at least in part—due to interference with these TNF-dependent microvascular processes.

In conclusion, we established an experimental approach that allows the visualization and quantitative analysis of pathological processes in the microvasculature of salivary glands in real-time. Employing this model, we identified key adhesion and signaling molecules controlling myeloid leukocyte trafficking and subsequent microvascular leakage in inflamed glands. Integrating mouse models of autoimmune diseases and cancer, bacterial and viral pathogens, or irradiation injury in this approach will enable us to delineate disease-specific molecular targets for novel therapeutic strategies in salivary glands pathologies.

## Data Availability Statement

The raw data supporting the conclusions of this article will be made available by the authors, without undue reservation.

## Ethics Statement

The animal study was reviewed and approved by the Regierung von Oberbayern (Maximilianstraße 39, 80538 München, Germany).

## Author Contributions

All authors were involved in drafting the article or revising it, critically for important intellectual content, and all authors approved the final version to be published. Study conception and design: BU and CR. Acquisition of data: BU, CB, and JD. Analysis and interpretation of data: BU, CB, JD, JL, MC, and CR. All authors contributed to the article and approved the submitted version.

## Funding

This study is supported by Collaborative Research Centre (CRC) 914 of Deutsche Forschungsgemeinschaft (DFG).

## Conflict of Interest

The authors declare that the research was conducted in the absence of any commercial or financial relationships that could be construed as a potential conflict of interest.
