# Supplementary material for: A Novel Experimental Approach for In Vivo Analyses of the Salivary Gland Microvasculature
Source: Front Immunol. 2021 Feb 17;11:604470. doi: 10.3389/fimmu.2020.604470 (PMC7925411; doi:10.3389/fimmu.2020.604470)
Supplement: Supplementary file 1 [file Table_1.pdf]

Supplementary Material

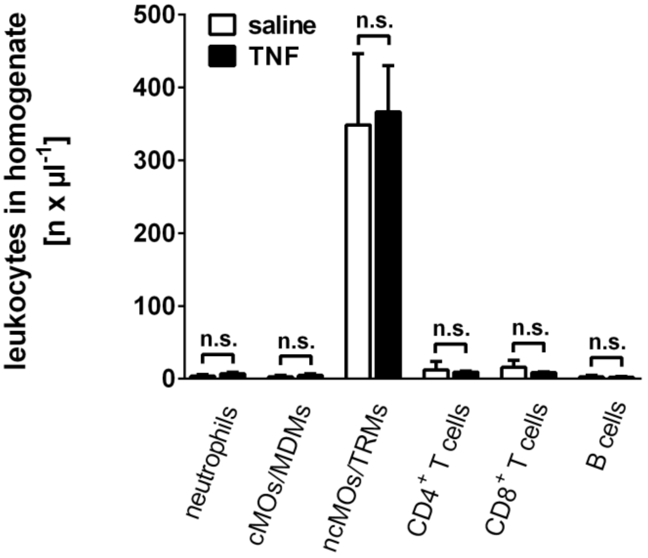

Supplementary Figure 1. Trafficking of leukocytes into the submandibular gland upon low dose TNF stimulation. Quantitative data on the recruitment of leukocyte subsets into submandibular glands upon superfusion with recombinant mouse TNF (0.5 ng/ml; 5 hours) or saline (mean ± SEM for n = 6; * $p < .05$ vs saline; n.s., not significant).

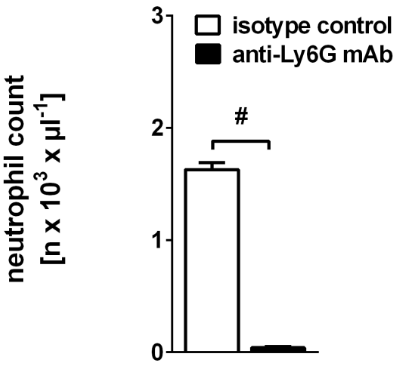

Supplementary Figure 2. Neutrophil blood counts upon antibody-mediated neutrophil depletion. Quantitative data on the neutrophil blood counts (IDEXX ProCyte Dx Hematology Analyzer) in animals treated with a depleting monoclonal anti-Ly6G antibody (clone: 1A8) or the isotype control antibody (mean ± SEM for n = 3; # $p < .05$ vs isotype control).
